# Heterologous expression of leader-less *pga *gene in *Pichia pastoris*: intracellular production of prokaryotic enzyme

**DOI:** 10.1186/1472-6750-10-7

**Published:** 2010-02-03

**Authors:** Helena Marešová, Zdena Marková, Renáta Valešová, Jan Sklenář, Pavel Kyslík

**Affiliations:** 1 Laboratory of Enzyme Technology, Institute of Microbiology, vvi, Academy of Sciences of the Czech Republic, Vídeňská 1083, 142 20 Prague 4, Czech Republic

## Abstract

**Background:**

Penicillin G acylase of *Escherichia coli *(PGA_Ec_) is a commercially valuable enzyme for which efficient bacterial expression systems have been developed. The enzyme is used as a catalyst for the hydrolytic production of β-lactam nuclei or for the synthesis of semi-synthetic penicillins such as ampicillin, amoxicillin and cephalexin. To become a mature, periplasmic enzyme, the inactive prepropeptide of PGA has to undergo complex processing that begins in the cytoplasm (autocatalytic cleavage), continues at crossing the cytoplasmic membrane (signal sequence removing), and it is completed in the periplasm. Since there are reports on impressive cytosolic expression of bacterial proteins in *Pichia*, we have cloned the leader-less gene encoding PGA_Ec _in this host and studied yeast production capacity and enzyme authenticity.

**Results:**

Leader-less *pga *gene encoding PGA_Ec_under the control of AOX1 promoter was cloned in *Pichia pastoris *X-33. The intracellular overproduction of heterologous PGA_Ec_(hPGA_Ec_) was evaluated in a stirred 10 litre bioreactor in high-cell density, fed batch cultures using different profiles of transient phases. Under optimal conditions, the average volumetric activity of 25900 U l^-1 ^was reached. The hPGA_Ec _was purified, characterized and compared with the wild-type PGA_Ec_. The α-subunit of the hPGA_Ec _formed in the cytosol was processed aberrantly resulting in two forms with C- terminuses extended to the spacer peptide. The enzyme exhibited modified traits: the activity of the purified enzyme was reduced to 49%, the ratios of hydrolytic activities with cephalexin, phenylacetamide or 6-nitro-3-phenylacetylamidobenzoic acid (NIPAB) to penicillin G increased and the enzyme showed a better synthesis/hydrolysis ratio for the synthesis of cephalexin.

**Conclusions:**

Presented results provide useful data regarding fermentation strategy, intracellular biosynthetic potential, and consequences of the heterologous expression of PGA_Ec _in *P. pastoris *X-33. Aberrant processing of the precursor of PGA_Ec _in the cytosol yielded the mature enzyme with modified traits.

## Background

Bacterial expression systems enjoy great popularity because of their well-characterized genetics, availability of "taylor-made" cloning vectors as well as hosts, and ability to grow rapidly on inexpensive substrates. These systems are frequently used to construct cell factories for the expression of homologous or heterologous proteins and many industrial production strains were prepared in this way.

Penicillin G acylase (PGA, EC 3.5.1.11) is an excellent example of the commercially valuable enzyme for which a large number of bacterial expression systems was developed [1,2].

The PGAs of *E. coli*, *Arthrobacter viscosus*, *Providencia rettgeri*, *Kluyvera citrophila*, *Bacillus megaterium *and *Alcaligenes faecalis *have been described [3-8] and studied in details regarding the production of β-lactam nuclei by N-deacylation of penicillin G or deacetoxycephalosporin G. The same PGAs can be used as potential catalysts for the kinetically controlled synthesis of semi-synthetic antibiotics such as ampicillin, amoxicillin, cephalexin, or cefadroxil from the β-lactam nucleus and appropriate activated acyl donor. The efficiency of the synthetic process is generally quantified by the ratio of the rates for the synthetic reaction and hydrolysis of activated acyl donor (ratio S/H): the enzymes with higher value of this parameter are more convenient for a synthetic use [9-11].

Experimental data on the expression of *pga *gene and posttranslational processing of PGA were obtained predominantly from studies on the PGAs of enterobacteria: the enzyme accumulates in the periplasmic space as a heterodimer and, to become a mature, properly folded enzyme, the precursor peptide has to undergo rather complex processing. The PGA precursor is an inactive prepropeptide that consists of a signal (leader) peptide and a propeptide (spacer) separating α and β-subunits. Processing of the enzyme precursor in *E. coli *begins in the cytoplasm where the β-subunit is released by intra-molecular autocatalytic cleavage [12]. The signal sequence of the precursor is cleaved upon crossing the cytoplasmic membrane which is followed by an intermolecular, sequential removing of the propeptide generating the C-terminus of the α-subunit [13,14]. The PGA from gram-positive bacteria such as *B. megaterium *or *A. viscosus *is secreted to the growth medium [4,15].

The expression system based on the methylotrophic yeast *Pichia pastoris *is another excellent cell factory based on eukaryotic microorganism: the system competes with the prokaryotic one in many aspects, e.g., stable genomic integration of the recombinant expression cassette, high-cell density cultivation, and efficient secretion of the product [16,17]. Although the system is predominantly used as a secretory system, efficient cytosolic expression can be achieved. The data on the intracellular expression of bacterial proteins are scarce [18,19]. In the case of prokaryotic esterase of *Lactobacillus casei*, cytosolic expression in *Pichia *was found to be superior to a prokaryotic expression system: the expression of the esterase was as much as 50-fold higher in *P. pastoris *X-33 than in *E. coli *BL2 [19].

The PGA of *E. coli *was expressed in *Saccharomyces cerevisiae*: the *pga *gene was cloned on a multicopy plasmid and the enzyme was secreted to the medium in small amounts [20]. The PGA from *P. rettgeri *secreted by the same host was partially O-glycosylated at the α-subunit [21]. This PGA was also cloned and expressed in *P. pastoris *[22,23]. The secreted enzyme was entirely N-glycosylated, functional, and exhibited higher thermostability [22]. The extracellular production in a stirred bioreactor reached the volumetric and specific activities of 26500 U l^-1 ^and 18 U mg_protein_^-1^, respectively [23].

So far, the only way of preparation of PGA for industrial use is the expression of the enzyme in prokaryotic hosts. The production capacity for non-glycosylated prokaryotic PGA in *Pichia *cytosol could be higher but so far this approach has not yet been used.

We have cloned the leader-less gene encoding PGA from *E. coli *(PGA_Ec_) under the control of AOX1 promoter in *P. pastoris *X-33. Intracellular expression of the gene was studied in a stirred bioreactor in a high-cell density culture under different profiles of the transient phase applied in the fed-batch process. The heterologous PGA_Ec _(hPGA_Ec_) was purified, characterized and compared with the wild-type PGA_Ec _regarding propeptide processing, substrate specificity, physico-chemical traits, kinetic parameters, and kinetically controlled synthesis of β-lactam antibiotics.

## Results

### Cloning of the leader-less *pga *gene

The structural gene for PGA_Ec _(2541 base pairs (bp), including stop codon) encodes the preproprotein with 846 amino acids (aa). Chromosomal DNA from the strain *E. coli *RE3 was used in PCR reactions to obtain the leader-less *pga *gene starting from the 79^th ^bp after ATG triplet. In order to express the *pga *gene in *P. pastoris *X-33, the upstream PCR primer contained a yeast consensus sequence (with ATG triplet) followed with the first triplet of the α-subunit. This construct was inserted into the pPICZ-A under the control of AOX1 promoter and the plasmid was used to transform the host *P. pastoris *X-33. Zeo^R ^clones identified on selective plates were cultured in shake flasks with methanol and tested for the PGA activity: among 30 clones with Mut^+ ^phenotype, 19 isolates expressed elevated amounts of the PGA ranging from 0.18 to 2.6 U ml^-1 ^after 64 h of induction. The Mut^+ ^isolate with the highest activity was designated as *P. pastoris *X-33(pPIC-PA1) and selected for further work.

### High-cell density culture

The expression of leader-less *pga *gene of *E. coli *by eukaryotic system of *P. pastoris *X-33(pPIC-PA1) was studied in a standard, four phase process in a stirred bioreactor. To evaluate the maximum synthetic capacity of the yeast system for the intracellular expression of hPGA_Ec_, a transient phase of the process was optimized (the phase required for the transition of cell metabolism from glycerol to methanol in terms of concentration of the methanol as an inducer and profile of the carbon source supplementation).

Four experiments were designed: the cultures with transient profile I (A and B) where continuous induction with methanol (0.5 or 1.5%) was accompanied with linear drop of glycerol feeding to zero; the cultures with the profile II (C and D) in which the cultures were starved for glycerol for two hours, and the induction was started with the dose of methanol (1.5% or 0.5%) at the end of the transient phase. In all experiments, the feeding rate of methanol at the methanol feed phase was controlled by dissolved oxygen concentration: the process values of pO_2 _ranged from 5 to 30%.

**Figure 1:**
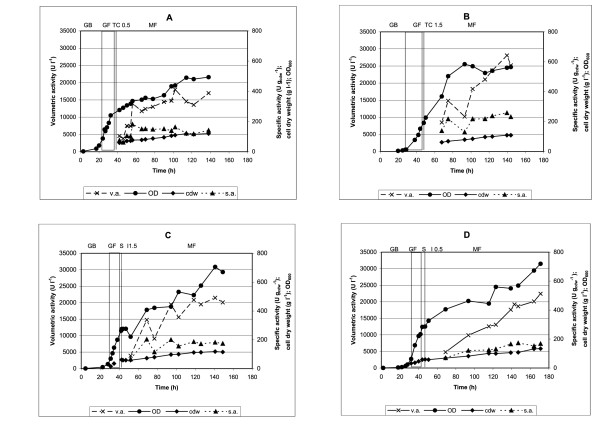
**The effect of transient phase profile on hPGA_Ec _production in fed-batch cultures of the strain *P. pastoris *X-33(pPIC-PA1)**. Time courses of the values of the cell dry weight (g l^-1^), optical density (OD_600_), volumetric activity (U l^-1^) and specific activity of hPGA_Ec _(U g_cdw_^-1^) assayed in the cultures in a 10- l stirred bioreactor. Phases of the cultures: GB, glycerol batch culture; GF, glycerol feed culture; TC, transient phase with linear decrease of glycerol concentration to 0 and constant feed of methanol (A- final concentration of methanol 0.5%, B- final concentration of methanol 1.5%); SI, transient phase with starvation for glycerol and single dose of methanol at the end of the phase (C- final concentration of methanol 1.5%, D- final concentration of methanol 0.5%); MF, methanol feed culture.

Samples were taken at regular intervals and the courses of the culture parameters (values of the volumetric activity, specific activity, and cell dry weight concentration) and calculated average final parameters of the cultures are shown in Figure 1 and Table 1.

**Table 1:** The effect of the transient phase profile on parameters of the fed-batch culture of *P. pastoris *X-33(pPIC-PA1) in a 10-l stirred bioreactor

TP profile - Experiment	Methanol	Maximal volumetric activity (h)*	Specific activity	Cell dry weight	Average volumetric activity	Average cell dry weight	Induction time	Specific productivity
	**(%)**	**(U l^-1^) (h)**	**(U g_cdw_^-1^)**	**(g l ^-1^)**	**(U l^-1^)**	**(g l ^-1^)**	**(h)**	**(U g_cdw_^-1 ^h^-1^)**

I - A	0.5	18000 (102)	163	120	16000	120	79	1.68
I - B	1.5	28100 (140)	258	109	25900	106	73	3.34
II - C	1.5	21500 (140)	182	115	20510	115	63	2.8
II - D	0.5	22420 (170)	167	130	20400	124	100	1.64

TP profile - transient phase profile: I - linear feeding of methanol, linear decrease of glycerol feeding, II - starvation for glycerol; methanol supplemented at the end of the transient phase in a single dose. Methanol - final concentration at the beginning of methanol feeding phase. * (h) means the hour at which maximal volumetric activity was achieved. Average volumetric activity - volumetric activity calculated from the last three values of activity assay. Induction time - time of growth on methanol required to achieve the average activity.

The highest specific productivities of the hPGA_Ec _3.34 and 2.8 U g_cdw_^-1 ^h^-1 ^were achieved in the experiments B and C, respectively: the inducer concentration in these cultures equalled 1.5%. In addition, the highest average volumetric activity (25900 U l^-1^) and specific activity (258 U g_cdw_^-1^) were reached in the experiment B (the transient phase profile I).

### Purification of PGA from *E. coli *and *P. pastoris *and determination of authenticity of the heterologous product

**Figure 2:**
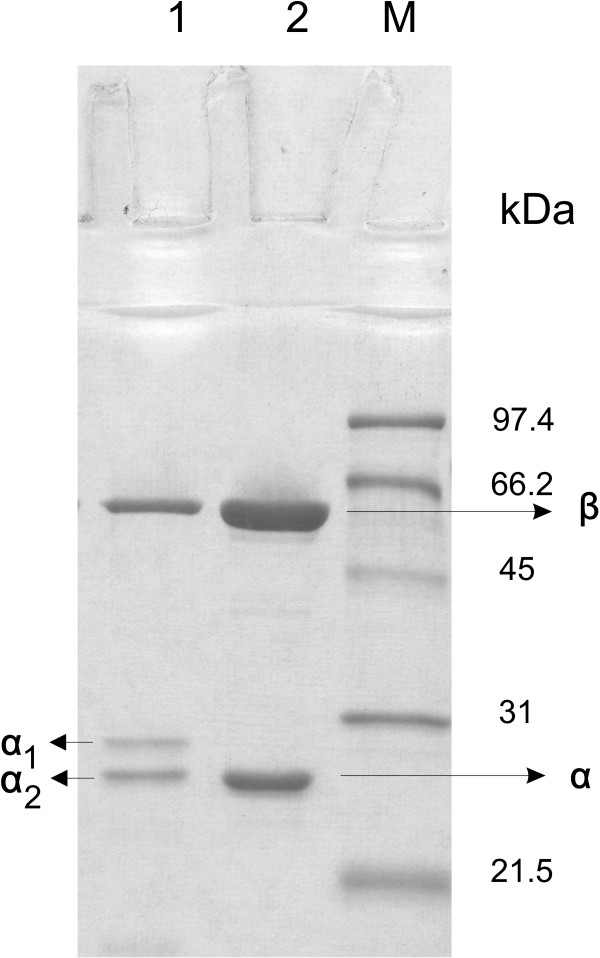
**Comparison of the subunits of PGA_Ec_and hPGA_Ec_**. SDS-PAGE analysis of the purified hPGA_Ec _expressed in *P. pastoris *X-33(pPIC-PA1) (lane 1) and PGA_Ec _purified from *E. coli *RE3(pKA18) (lane 2). Low molecular mass standard (lane M). The gel was stained with Coomassie blue.

To characterize both PGAs, we purified the wild type PGA from the strain *E. coli *RE3(pKA18) and hPGA_Ec _from *P. pastoris *X-33(pPIC-PA1). The PGA_Ec _from the prokaryotic host was purified by a purification process consisting of the disintegration of cells, preparation of crude enzyme solution, fractional precipitation, gel filtration, and ion exchange chromatography. The yield of the activity was 20%, the enzyme was purified 29-fold and had the activity of 64.6 U mg_protein_^-1^. Purity control of the isolated PGA_Ec _by SDS-PAGE electrophoresis revealed two protein bands with molecular weights corresponding to α and β-subunits. Calculated molecular weights from MALDI-TOF MS data of PGA_Ec _were 23.8 and 62.4 kDa for α and β-subunits, respectively, and corresponded to theoretical values of 23.8 and 62.4 kDa (Figure 2).

To isolate the hPGA_Ec _from *P. pastoris *X-33(pPIC-PA1), crude enzyme solution was prepared by disintegration of the biomass from the fed-batch experiment B. The inhibitors of proteases in the course of hPGA_Ec _purification were used to exclude any further non-specific proteolysis of the enzyme.

SDS-PAGE gel analysis after each purification step showed that the enzyme was sufficiently pure after the purification step on DEAE fractogel. The enzyme was purified 26-fold with an overall activity yield of 46% and had the activity of 31.8 U mg_protein_^-1^. SDS-PAGE gel electrophoresis revealed presence of two forms of the α-subunit: α_1 _(36%) and α_2 _(64%). Molecular weights calculated from MALDI-TOF MS data were 24.0, 25.9 and 62.4 kDa for α_2_, α_1_, and β-subunit, respectively (Figure 2).

Comparison of MALDI-TOF MS analysis of tryptic digests of the α-subunit from *E. coli *and α_1_, α_2_-subunits from *P. pastoris *X-33 revealed differences in primary structures: the C-terminus of the α_2_-subunit extends to the amino acid residue A_210 _of the spacer peptide (extension by 1 aa) and the C-terminus of the α_1 _extends to K_227 _of the spacer (extension by 18 aa). The C-terminus of the α-subunit from PGA_Ec _is formed by A_209_. The N-terminus was investigated according to the modified protocol based on the use of the reagent 4-sulfophenyl-isothiocyanate (SPITS) before protein separation by SDS-PAGE electrophoresis. Analysis of the labelled N-terminus confirmed that the terminuses of α_1 _and α_2_-subunits of hPGA_Ec _start with methionine which is missing in the mature form of PGA_Ec_. As regards the β-subunits of PGA_Ec _and hPGA_Ec_, they were identical.

### Physico-chemical and kinetic parameters of PGA_Ec _and hPGA_Ec_

**Figure 3:**
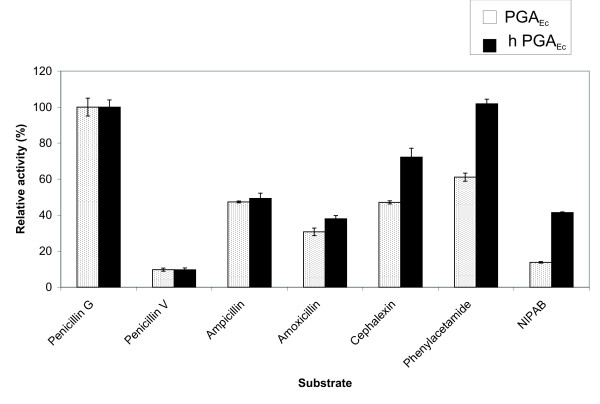
**Comparison of substrate specificities of PGA_Ec _and hPGA_Ec_**. Relative activities (%) of the purified PGAs with different substrates are shown. The activities of both PGAs measured with penicillin G as reaction substrate were taken as 100%. Three parallel measurements were performed with the same enzyme sample.

Hydrolytic activities of both purified PGAs were determined with penicillin G, penicillin V, ampicillin, amoxicillin, cephalexin, 6-nitro-3-phenylacetylamidobenzoic acid (NIPAB), and phenylacetamide (Figure 3). It is evident that the enzymes differ in the substrate specificity: in terms of a hydrolytic activity ratio, they have the same values for penicillin V, ampicillin or amoxicillin to penicillin G. The increased ratio of the hydrolysis of cephalexin, phenylacetamide or NIPAB to penicillin G was observed with hPGA_Ec _and the value ranged from 50% (cephalexin) to 200% (NIPAB).

Stability of the hydrolytic activity (the effect of the temperature or pH), temperature optimum and pH optimum of the enzyme were determined for both enzymes with penicillin G as the reaction substrate. It was found that PGA_Ec _exhibited high stability in the range of pH from 3 to 8.5, while hPGA_Ec _is stable only in the range of pH from 4 to 8. The activity of the heterologous enzyme dropped by 15-20% at pH 3.5 and 8.5. The remaining activity of 50% was assayed at pH values 3 and 9.

The effect of the temperature on the stability of the enzymes was studied in the range of temperatures from 20 to 70°C. Both enzymes were stable up to 40°C. At higher pre-incubation temperatures, the remaining activities of both enzymes fell down rapidly.

Determination of the pH and temperature optima suggested that the temperature and pH optimum for the hydrolytic activity of PGA_Ec _and hPGA_Ec _were 55°C, pH 8.0 and 50°C, pH 7.5, respectively.

The kinetic parameters were determined with the natural substrate penicillin G and chromogenic substrate NIPAB. The initial reaction rate of the hydrolysis of the substrate was measured in the range of substrate concentrations: 5-100 μM for penicillin G and 7-2000 μM for NIPAB. The values of *K*_m_, *k*_cat _and *V*_max _of PGA_Ec _and hPGA_Ec _are shown in Table 2.

**Table 2:** Kinetic parameters of PGA_Ec _and hPGA_Ec_

Enzyme	Substrate	*K_m_*	*V*_max_	*k_cat_*
		(μM)	(μmol min^-1 ^ml^-1^)	(s^-1^)
PGA_Ec_	penicillin G	34	121	13.6
	NIPAB	28	15	1.6
hPGA_Ec_	penicillin G	12	7.7	6.4
	NIPAB	30	2.6	2.2

To check if the aberrant processing of hPGA_Ec _could result in improved traits of the enzyme in syntheses of β-lactam antibiotics, kinetically controlled syntheses of cephalexin, amoxicillin and ampicillin catalyzed by both purified PGAs were carried out at pH 7.0 and 30°C. We found that the S/H ratios for the enzymatic formation of cephalexin by PGA_Ec _and hPGA_Ec _were 2.6 and 3.9, respectively. Both enzymes PGA_Ec _and hPGA_Ec _have similar S/H ratios for the synthesis of ampicillin from 6-aminopenicillanic acid (6-APA) and D-phenylglycine amide (1.7 and 1.9), and amoxicillin from 6-APA and D-p-hydroxyphenylglycine amide (1.1 and 1.0).

## Discussion

At present, production strains for PGA used in industry are mainly the recombinant strains of *E. coli *based on a homologous cloning of the genes: the same microorganism is used as the *pga *gene donor and the host for the recombinant plasmid. The finding that penicillin acylases may also catalyze syntheses of β-lactam antibiotics (e.g., ampicillin, amoxicillin, cephalexin etc.) started the search for "novel" or "improved" penicillin acylases with more convenient traits for these synthetic applications [24]. The development of the eukaryotic expression system based on *P. pastoris *and "bio-sourcing" of the genes from the environmental DNA revealed an underestimated aspect of the expression of the cloned gene in a foreign host: an authenticity of the heterologous protein.

To evaluate the production potential of *P. pastoris *X-33 for intracellularly expressed PGA*_Ec_*, the leader-less *pga *gene was integrated into the chromosome of *P. pastoris *X-33 and the Mut^+ ^strain *P. pastoris *X-33(pPIC-PA1) was constructed. The gene was not adapted to *P. pastoris *codon usage and its intracellular expression induced with methanol was studied in a stirred bioreactor in high-cell density cultures. The effect of the profile of the transient phase of the fed-batch culture on penicillin acylase production was selected as the parameter for the optimization of the fermentation process. It can be concluded that the change of glycerol to methanol utilization during the transient phase had a marked effect on the volumetric activity and specific productivity of the culture: a continuous decrease of glycerol accompanied with linear increase of the methanol concentration (up to 1.5%) resulted in the highest values of both parameters.

When the production capacity for the homologous PGA_Ec _was studied in a fed-batch culture of the recombinant *E. coli*, the specific activity of about 900 U g_cdw _^-1 ^was reached [25] which corresponded to the volumetric activity of about 14000-22000 U l^-1 ^(the scale-up of fermentation to a 300-l bioreactor) [26]. In our experiments, the volumetric activity of hPGA_Ec _expressed in *P. pastoris *X-33 after induction with 1.5% methanol reached higher level and ranged from 25000 to 28000 U l^-1^. However, the specific activity was lower in comparison with the bacterial culture.

The finding that the activity of the purified enzyme hPGA_Ec _is low (about 31 U mg_protein_^-1^) was surprising. Considering the pattern of the subunit bands on SDS gel, we have concluded that hPGA_Ec _is not correctly maturated in the cytosol of *P. pastoris *X-33 and the enzyme is a mixture of two isoforms in the ratio 1(α_1_β) to 2(α_2_β). As expected, we have proved by deglycosylation experiment (data not shown), that no N-glycosylation occurred as it was described for the secreted enzyme of *P. rettgeri *in *P. pastoris *GS115 [22]. The authors reported 3 types of the β-subunit and 2 forms of the α-subunit: while the β-subunit was glycosylated to a different extent (three bands), two bands of the α-subunit were formed due to non-specific proteolysis at the N-terminus of the subunit. However, the C-terminus of the smaller subunit was not analyzed.

In our case, different processing occurred in the cytosol at this very terminus. Amino acid sequence analysis of the N- and C-terminuses confirmed that the β-subunits of PGA_Ec _and hPGA_Ec _are identical and that the first step of the precursor maturation, an autocatalytic cleavage between the spacer peptide and β-subunit, occurs correctly in the cytosol. This finding is in agreement with autocatalytic intracellular processing and the N-terminus formation of the β-subunit found in prokaryotes [14].

At present, we have no piece of evidence if the host- or non-specific proteolysis is involved in processing of the hPGA_Ec _precursor consisting of the α-subunit and propeptide. It is known that the α-subunit of PGA is responsible for the substrate specificity of the enzyme [27]. Modified primary structures of the α-subunits can explain the lower activity of purified hPGA_Ec _expressed in the cytosol of *P. pastoris *X-33: the α-subunits start with methionine and their C-terminuses extend to A_210 _of the spacer peptide (extension by 1 aa, α_2_-subunit) or K_227 _(extension by 18 aa, α_1_-subunit).

In terms of biosynthetic potential of the cell for the properly matured enzyme, the volumetric activity and specific activity of hPGA_Ec _would correspond to about 52000 U l^-1 ^and 516 U g_cdw_^-1^, respectively, because the ratio of the activity of purified PGA_Ec _to hPGA_Ec _equals about two.

A presence of several mature forms of the periplasmic PGA from *A. faecalis *expressed in *E. coli *was also described [28] and the results were discussed in terms of the conversion of the N-terminal glutamine to pyrrolidone carboxylic acid and the loss of three aa from the C-terminus of the α-subunit.

It is evident [[22,23,28] our results], that heterologous expression of PGA may yield a mature enzyme with modified primary structure and traits.

A hydrolysis of selected substrates is an important parameter for evaluation of any changes in the substrate specificity of the heterologous enzyme that may have occurred at the expression in a given host. The hPGA_Ec _has got higher affinity to cephalexin (substrate with 7-aminodeacetoxycephalosporanic acid moiety, 7-ADCA), phenylacetamide and NIPAB (a chromogenic synthetic substrate based on nitrobenzoic acid). There was no change in the enzyme affinity to the substrates containing 6-APA moiety. Regarding the kinetic parameters determined with the natural substrate penicillin G, aberrant maturing of hPGA_Ec _resulted in lower *K*_m_, *V_max_*and *k_cat_*.

Compared to the properly matured enzyme, the S/H ratio of hPGA_Ec _for the synthesis of cephalexin was increased by 50%. Improvement of PGA traits as regards the synthesis of cephalexin was described for a chimeric penicillin acylase. After shuffling of the *pga *genes of *E. coli*, *P. rettgeri *and *K. cryogenes*, the mutant enzyme displayed a 40% higher synthetic activity than the wild type enzyme of *P. rettgeri *[29].

The pH and temperature stabilities of both enzymes are comparable with literature data [30,31] and the heterologous expression in *P. pastoris *X33 system has no marked effect on these parameters.

## Conclusions

The prokaryotic, leader-less gene encoding PGA_Ec _under the control of AOX1 promoter can be efficiently expressed in the cytosol of the yeast *P. pastoris *X-33. The activity of the mature enzyme equals 31.8 U mg_protein_^-1 ^and this value corresponds to 49% of the PGA_Ec _activity. It was shown that the precursor of hPGA_Ec _was processed aberrantly and the enzyme exhibited improved traits for the synthesis of cephalexin, a β-lactam antibiotic containing 7-ADCA moiety. No improvement was observed in syntheses of the antibiotics derived from 6-APA. The understanding on structure/function relationships of the aberrantly processed or engineered propeptide of a penicillin acylase could be applicable to modify the traits of the enzyme.

## Methods

### Strains and expression constructs

The construction of the strains *E. coli *RE3 (CCM 4228) and RE3(pKA18) have been described earlier [2,32].

The leader-less *pga *gene was amplified in two steps by two PCR using the chromosomal DNA of the strain *E. coli *RE3 as a template. An upstream primer 5'-AGCGGTGAATAAAGCGATTCGTTT derived from a flanking part of 5'end of *pga *gene and a downstream primer 5'-TTTC**GGGCCC**AATTATCTCTCTGAAC were used in the first PCR reaction (the *Apa*I restriction site is highlighted in bold). The specific product from the first PCR reaction was used as a template in the second PCR reaction with pair of primers: upstream primer 5'-**ATAATG**GAGCAGTCGTCAA-GTGAG (a part of yeast consensus sequence is highlighted in bold) and the same downstream primer as in the first PCR reaction. The specific PCR product was cloned into *Pml*I/*Apa*I restriction sites of the vector pPICZ-A (Invitrogen). The constructed plasmid was designated as pPIC-PA and used to transform the host *E. coli *TOP10. Plasmid bearing the leader-less *pga *gene from Zeo^R ^transformant was linearized by *Sac*I and transformed by electroporation into the host *P. pastoris *X-33. All routine techniques for DNA manipulations were performed as described previously [33].

### Media and flasks cultures

*E. coli *strains were cultured in low-salt Luria-Bertani (LB) medium at 37°C. If required, Zeocin (25 μg ml^-1^) was supplemented. *P. pastoris *X-33 was cultured in YP medium (1% yeast extract, 2% peptone) supplemented with 2% glucose (YPD medium) or 2% glycerol (YPGly medium). Solid YPDS medium (1% yeast extract, 2% peptone, 2% glucose, 1 M sorbitol, 2% agar, and 100, 200, 500 or 1000 μg of Zeocin per ml) was used for selection of the yeast transformants.

### Screening for Zeo^R ^positive clones

A screening was carried out on solid YPDS medium supplemented with Zeocin (100 μg ml^-1^) after incubation for 3 days at 28°C. Subsequent selection of 150 Zeo^R ^isolates was carried out using replica plating on solid YPD medium containing 200, 500 or 1000 μg ml^-1 ^of Zeocin. Thirty clones from the plates with the highest concentration of Zeocin were grown in 10 ml of YPGly medium with biotin (4 × 10^-5 ^%) for 16 h at 28°C. Each culture was centrifuged at room temperature, the cell pellet was resuspended in 20 ml of YP medium to get OD_600 _of 1.0 and methanol was added to a final concentration of 0.5% to induce the enzyme synthesis. Every 24 hours, an equal dose of methanol was added to maintain the culture growth for 64 hours. Cultures were sampled at given intervals for the enzyme assay.

### High-cell density culture in stirred bioreactor

The fed-batch culture was carried out in a stirred bioreactor Biostat MD (B. Braun Biotech International, Melsungen, Germany) with initial working volume of 6 litres at 28°C. The batch culture medium contained per litre: 6 g KOH, 10 g MgSO_4_.7H_2_O, 1.3 g KI, 40 ml glycerol and 10 ml of 85% H_3_PO_4_. Solution of CaSO_4 _(1.3 g) and biotin (0.4 mg l^-1 ^of medium) were sterilized separately. PTM1 trace salt stock solution contained per litre: 65 g FeSO_4_.7H_2_O, 6 g CuSO_4_.5H_2_O, 3 g MnSO_4_.H_2_O, 20 g ZnCl_2_, 0.2 g Na_2_MoO_4_.2H_2_O, 0.8 g KI, 0.2 g H_3_BO_3_, 0.5 g CaSO_4_.2H_2_O, 5 ml of concentrated H_2_SO_4_, and was added to the medium after sterilization (2 ml per litre of the medium). Two feeding solutions were prepared: glycerol (50% water solution) and methanol (99.5% solution). Both solutions were supplemented with trace metals and biotin in amounts stated above. The airflow was kept constant at the maximum of the system (10 litres of air per minute) and the pH of the culture was controlled at 5.0 by NH_4_OH (25% water solution). A concentration of dissolved oxygen (pO_2_) was maintained at 30% of the value for air saturation of the medium by cascade regulation of stirring frequency in the course of the initial batch phase. The glycerol as well as methanol feeding was controlled by the value of pO_2 _that was set up to the value of 30%.

The flask inoculum (10 ml of YPGly medium) was inoculated with a single colony from agar plate with YPD medium and cultured on orbital shaker for 20 hours at 28°C. Two ml of the culture were transferred to 100 ml of YPGly medium, the flask was cultured for 24 hours at 28°C and the whole culture was used to seed the medium in a bioreactor. A standard fed-batch process consisting of four phases was used to express the heterologous *pga *gene: phase 1 - a batch culture in the medium supplemented with glycerol (about 40 h); phase 2 - feeding of glycerol to reach high concentration of the biomass (OD of about 280); phase 3 - transition of the cell metabolism from glycerol to methanol (90 - 120 min), and phase 4 - induction of the enzyme expression by feeding of methanol.

To maximize the production of hPGA_Ec_, two strategies dealing with the phase 3 were applied. Profile I: a linear increase of methanol concentration in the course of the transition phase up to 0.5 and 1.5% in experiment A and B, respectively, accompanied simultaneously with linear decrease of glycerol feeding rate to zero. Profile II: the culture was starved in the course of the transient phase both for glycerol and methanol; after 120 min, methanol was added in a single dose to reach final concentration of 1.5 and 0.5 % in experiment C and D, respectively.

### Assay of hydrolytic activity

The biomass from 1-ml samples of a culture was separated by centrifugation and rinsed with distilled water. The pellet was resuspended in 1 ml of 0.1 M sodium phosphate buffer (pH 8.0), 1.25 ml of glass beads were added (diameter of 0.5 mm, Willy A. Bachofen AG. Basel, Switzerland), and the cells were mechanically disrupted by vortexing for 20 min. The debris of cells was removed by centrifugation and the activity of PGA was measured in the supernatant. The activity of PGA was assayed in 0.05 M sodium phosphate buffer (pH 8.0) supplemented with penicillin G (2% solution) using the method described by [34]. The activity of one unit (U) is defined as the amount of PGA producing 1 μmol of 6-APA min^-1 ^at 37°C. The specific activity is expressed in U per g of cell dry weight (cdw).

Hydrolysis of substrates (penicillin G, penicillin V, ampicillin, amoxicillin, cephalexin, NIPAB and phenylacetamide; concentration of 0.5%) was carried out in 50 mM phosphate buffer, pH 7.5 (hPGA_Ec_) or 8 (PGA_Ec_) at 37°C. The resulting 6-APA and 7-ADCA were monitored by spectrophotometer at 415 nm after coupling with p-dimethylaminobenzaldehyde [34]. Determination of the activity with phenylacetamide was based on the measurement of liberated ammonia [35]. Hydrolysis of NIPAB was measured by spectrophotometer according to [3]. The molar extinction coefficient for 3-amino-6-nitrobenzoic acid (ε_405_) equalled 9.09 mM^-1^cm^-1^. The activity of one unit (U) was defined as the amount of the enzyme that hydrolyzed 1 μmol of NIPAB per min in 50 mM phosphate buffer, pH 7.5 (hPGA_Ec_) or 8 (PGA_Ec_) at 37°C.

### Enzyme purification

Biomass (50 g wet weight) of the strain *E. coli *RE3(pKA18) from 2 l of fermentation broth obtained as described earlier [32] was disintegrated and cell-free enzyme extract was prepared by thermal treatment in the presence of polyethyleneimine (45°C, pH 5.0). PGA was precipitated with ammonium sulphate from 50 ml of the enzyme extract at concentration between 40-60% of sulphate saturation. The precipitate was collected by centrifugation (5000 g, 35 min, 4°C), dissolved in 10 mM phosphate buffer (pH 8.0), dialyzed against the same buffer and the enzyme solution was applied to a DEAE-fractogel EMD 6500(S) column. The elution was carried out with the same buffer. The fractions having the activity of PGA were pooled and dialyzed against 1 mM phosphate buffer (pH 7.0). The enzyme solution was applied to a hydroxyapatite column equilibrated with 1 mM phosphate buffer (pH 7.0) and PGA was eluted using a linear gradient 0-100% of 200 mM phosphate buffer. The pooled fractions were concentrated by utrafiltration on Amicon 8010 and washed with 150 mM NaCl in 50 mM phosphate buffer (pH 7.5). The enzyme solution was applied to a Superdex 200 column equilibrated with 50 mM phosphate buffer (pH 7.0) containing 150 mM NaCl. PGA was eluted from the column and the pooled fractions were ultrafiltrated on Amicon 8010, washed as described above and stored at -20°C.

The hPGA_Ec _from *P. pastoris *X-33(pPIC-PA1) was purified from 11.8 g wet weight of the biomass from the experiment B: the biomass was resuspended in 50 ml of 0.1 M sodium phosphate buffer (pH 8.0) with 0.5 ml of Protease Inhibitor Cocktail (Sigma). 50 ml of glass beads were added to disrupt the cells on Lab-Shaker (A. Kühner AG, Switzerland) at 330 rpm for 1 h. After centrifugation (10000 g, 20 min, 4°C), CYSEP 329 (CYTEC Industries BV, Netherlands) was added to the supernatant up to the final concentration of 7.5%. Precipitated bulk protein was separated by centrifugation and PGA in the supernatant was precipitated with ammonium sulphate (up to 50% of sulphate saturation). The aggregated PGA was collected by centrifugation (5000 g, 35 min, 4°C), dissolved in 10 mM phosphate buffer (pH 8.0), dialyzed against the same buffer and the enzyme solution was applied to a DEAE-fractogel EMD 6500(S) column. The elution was carried out with the same buffer and the fractions having the activity of PGA were pooled and ultrafiltrated on Amicon 8010.

Protein concentrations were determined by means of BCA Protein assay kit (Pierce, Rockford, Ill). Bovine serum albumin was used as a standard.

### MALDI-TOF Mass Spectrometry

The subunits of the purified PGA were separated by 12% SDS-PAGE electrophoresis. Coomassie Blue-stained protein bands were cut out and digested "in gel" with a sequencing grade trypsin (Promega). The generated peptides were extracted from the gel and measured by MALDI-TOF MS as described earlier [36]. Positive ion MALDI mass spectra were measured on a Bruker BIFLEX reflectron time-of-flight mass spectrometer (Bruker Daltonics, Bremen, Germany) equipped with a gridless delayed extraction ion source, and a nitrogen laser (337 nm). Instrument was calibrated externally with a PepMix calibration kit (Bruker Daltonics). Samples of peptides (1 μl) deposited and dried on target were overlaid with 1 μl of diluted matrix α-cyano-4-hydroxy-cinnamic acid (one volume of saturated solution in methanol mixed with two volumes of 50% methanol and 0.3% trifluoroacetic acid (TFA)). All the measured masses are monoisotopic M+H adduct ions.

For determination of the N-terminuses, proteins were labelled at their N-terminuses with 4-sulfophenyl-isothiocyanate reagent (10 mg ml^-1 ^of SPITC in 20 mM NaHCO_3_) using modified protocol [37]. 10 μl of the reagent were added to the dry protein. Immediately after 30 min incubation at 56°C, the loading sample buffer was added and the samples were treated according to the conventional protocol for SDS-PAGE electrophoresis. The separated proteins were digested with trypsin according to the standard protocol [36]. Before measurement the peptides were desalted as follows: the peptides were deposited on target, dried on air, overlaid with CCA matrix (5 mg ml^-1 ^in 0.1% TFA, 50% acetonitrile), and washed with 5 μl of 0.1% TFA for 5-10 s. The derivatized peptide was sequenced by MALDI TOF-TOF tandem mass spectrometry (Ultraflex III, Bruker Daltonics) to confirm the N-terminal sequence.

### Kinetic parameters determination and kinetically controlled syntheses

Kinetic parameters of PGAs for penicillin G were determined by titration of phenylacetic acid with 0.01 M NaOH using an autotitrator (Radiometer, Copenhagen, Denmark) at 37°C. Kinetic parameters for NIPAB were determined by spectrophotometer, as described above. The relationship between the initial rate of the reaction and substrate concentration (1-2000 μM) was determined using the Michaelis-Menten equation and a non-linear regression program (Enzfitter, Elsevier Biosoft). To calculate *k_cat _*of the purified hPGA_Ec_, molecular weights of 86.4 (the enzyme with α_2_) and 88.4 (the enzyme with α_1_) kDa in a ratio 2 to 1 were used. The densities of the subunit bands in SDS-PAGE gel were evaluated using Image analysis by Intelligent Quantifier program (BioImage, Ann Arbor, Mich).

A kinetically controlled enzymatic synthesis of cephalexin, ampicillin or amoxicillin was carried out according to [24]. The reaction was catalyzed by the purified PGAs in 0.05 M potassium phosphate buffer (pH 7.0) at 30°C. The initial concentration of activated acyl donor (D-phenylglycine amide, D-p-hydroxyphenylglycine amide) was 15 mM and the concentration of an appropriate β-lactam nucleophile (7-ADCA or 6-APA) was 25 mM. All reactants were monitored in time by HPLC analysis and the initial rates of formation of the antibiotics and the hydrolysis of acyl donors were determined. The S/H ratio was calculated from the initial rates measured in the experiment.

### Effect of pH and temperature on enzyme activity

The temperature optimum for the activity was determined by measuring penicillin G hydrolysis in the range of temperatures 20-60°C. The thermal stability of PGAs was determined by incubation of the purified enzymes for 30 min in the range of temperatures 20-70°C and assaying of the remaining activity at 37°C with penicillin G at pH optima 8.0 and 7.5 for PGA_Ec _and hPGA_Ec_, respectively. The effect of pH on the activity of the enzyme was determined with penicillin G as the substrate at 37°C in Britton-Robinson buffer (40 mM H_3_PO_4_, 40 mM acetic acid and 40 mM H_3_BO_3_, the required pH was adjusted by 10% NaOH) in the range of pH 4-10. The pH-stability was determined by incubation of the enzyme sample at a given pH (ranging from 3-10, Britton-Robinson buffer) for 60 min at room temperature. Remaining activity was assayed at 37°C with penicillin G at pH optima 8.0 and 7.5 for PGA_Ec _and hPGA_Ec_, respectively.

## Competing interests

The authors declare that they have no competing interests.

## Authors' contributions

HM and RV carried out the molecular genetic studies and participated in the experiments with bioreactor cultivations. ZM carried out the purification of enzyme. JS performed Maldi-TOFF MS. HM and PK designed the research and wrote the manuscript. All authors read and approved the final manuscript.
